# More Recent Studies on Fragrances

**DOI:** 10.1289/ehp.112-a865a

**Published:** 2004-11

**Authors:** Ladd W. Smith

**Affiliations:** Research Institute for Fragrance Materials, Inc., Woodcliff Lake, New Jersey, E-mail: ehp@rifm.org

In response to Curtis (2004), I would like to cite more recent studies by researchers at the Research Institute for Fragrance Materials, Inc. (RIFM) that address the health and environmental effects of fragrances.

The RIFM strives to be the international leader for the safe use of fragrance ingredients (Balk et al. 2004; Bickers et al. 2003a; Cadby et al. 2002b; Smith 2003) and has ongoing research programs in the areas of fragrance allergy, human health effects (Cadby et al. 2002a; Bickers et al. 2003b), respiratory safety (Isola et al. 2004; Smith et al. 2004), and environmental impact (Salvito et al. 2002, 2004). The RIFM’s comprehensive, logical, and documented research methods are modeled after the National Academy of Sciences’ (NRC) Elements of Risk Assessment and Risk Management (NRC 1994).

Research is prioritized (Ford et al. 2000) and designed using information in the RIFM proprietary database (RIFM 2004) and according to the needs of the scientific community and the general public. The database provides a clearinghouse for human health and environmental studies, as well as basic material information, and is maintained by continuously monitoring journals, government reports, company-sponsored research, and available literature to enable analysis of documented conclusions.

All available information pertaining to the safety of fragrance materials, study protocols, and results are reviewed by an independent international panel of scientific and medical experts from the fields of toxicology, dermatology, pathology, and environmental science. Research results and safety evaluations are published in peer-reviewed scientific journals and presented at professional meetings.

In addition, the RIFM accepts proposals for sponsored scientific research and will work jointly with interested third parties to further knowledge on health and environmental issues.
